# TheGreater Bay Area: a new science powerhouse

**DOI:** 10.1093/nsr/nwaf533

**Published:** 2025-11-25

**Authors:** Hepeng Jia

**Affiliations:** Science communication at Soochow University and a freelance science writer for NSR

## Abstract

Heavy investments, novel institutions and intra-regional ties are fueling the scientific rise of the Guangdong–Hong Kong–Macao Greater Bay Area.

When Zhenglong Gu, then a professor of genetics at Ithaca, New York-based Cornell University, decided to return to China in 2021, he didn’t choose his hometown—the eastern Chinese Province of Jiangsu—or nearby big cities such as Shanghai or Hangzhou; nor did he go to Beijing, where he had his undergraduate studies. Instead, Gu came to the Greater Bay Area (GBA) Institute of Precision Medicine (Guangzhou) affiliated with Fudan University, where he initiated a Centre for Mitochondrial Genetics and Health Research.

‘As compared with those established institutions, here you can convene interesting young guys to do fascinating studies,’ says Gu.

His choice isn’t unique. The GBA has quickly grown to become one of China’s leading research hubs in recent years, having progressed from a relatively lagging position some 15 years ago.

**Figure:**
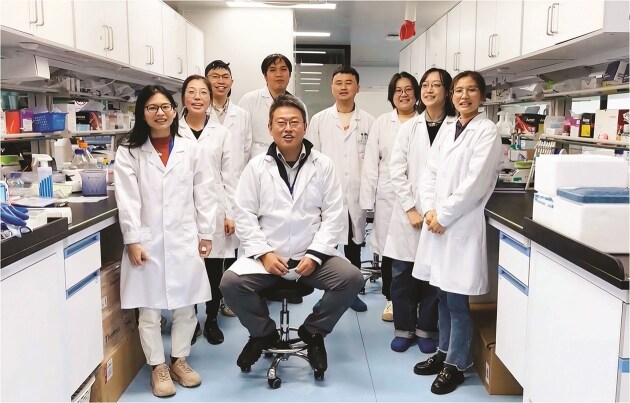
Zhenglong Gu (center) and his team in his lab at Fudan GBA Institute of Precision Medicine (Guangzhou) (Source: Zhenglong Gu)

## GBA’s DAZZLING EXPANSION IN SCIENCE AND TECHNOLOGY (S&T)

The Guangdong–Hong Kong–Macao Greater Bay Area, commonly referred to as the GBA, is a vast megapolitan region that encompasses nine major cities and two special administrative regions in southern China. Long regarded as one of China’s economic engines and a front line of reform and opening-up, the GBA has nonetheless lagged behind Beijing, Shanghai, Wuhan and Nanjing in the concentration of top-tier research institutions.

**Figure:**
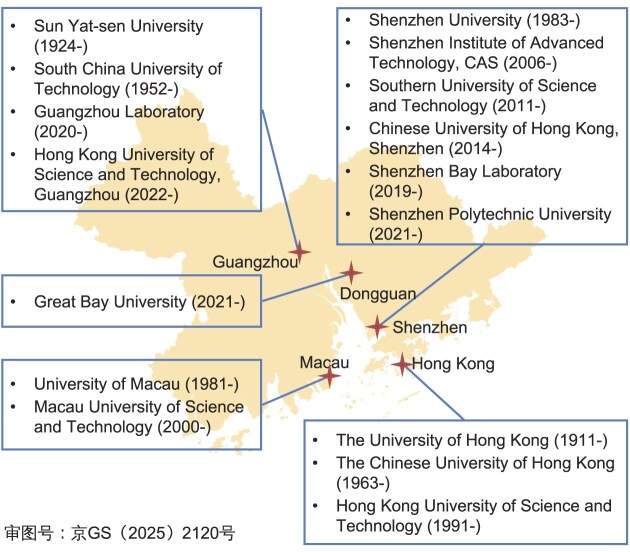
Representative research institutions in the GBA

Before GBA’s scientific surge, China’s research output was concentrated in Beijing and Shanghai, which hosted most institutes of the Chinese Academy of Sciences (CAS), as well as famous universities such as Peking University (PKU), Tsinghua University (THU), Fudan University and Shanghai Jiao Tong University (SJTU). They dominated China’s top-tier scientific outputs and received the greatest share of the funding from the National Natural Science Foundation of China (NSFC).

Guangzhou-based Sun Yat-sen University (SYSU) and South China University of Technology (SCUT), the only two nationally known universities in the GBA, had modest research outputs compared to the institutions in Beijing and Shanghai.

Although the central government’s plan to develop the GBA into an international center for science and technology innovation was only outlined in 2019, local governments—most notably Shenzhen’s—had already begun their pursuit of scientific excellence more than a decade earlier. In the late 2000s, Shenzhen was still largely a manufacturing hub with little academic presence. Hong Kong’s universities, such as the University of Hong Kong (HKU), ranked highly in Asia but lacked the research scale of leading institutions on the Chinese mainland. Meanwhile, Macao’s scientific output remained minimal.

According to the 2015 Nature Index, the GBA’s fractional count (FC), a metric that reflects a region or an institution’s contribution to high-quality scientific research by attributing authorship proportionally, was only around 200–300, significantly lower than that of Beijing (∼1200) and Shanghai (∼800), and was driven mainly by Hong Kong universities and SYSU.

But today, the Nature Index 2024 ranked the GBA among the top 10 global science cities, with an FC exceeding 600, surpassing many international hubs like Boston.

What drives the region to soar scientifically is not limited to the traditional leading universities like SYSU, but also the young institutions such as the Shenzhen-based Southern University of Science and Technology (SUSTech), which began enrolling students in 2012.

SUSTech produced 19 *Cell, Nature* or *Science* (CNS) papers in 2024, ranking 8th nationally—a remarkable feat for a young university. In April and May 2025 alone, Ning Jia, an associate professor at SUSTech, and colleagues published two papers in *Science*, both uncovering the clever ways bacteria defend themselves against viruses and how viruses fight back. In the first study [Hayes VM *et al. Science* 2025; adr3656], they found that some viruses produce tiny RNA molecules that mimic bacterial CRISPR guides. These ‘imposters’ sneak into the CRISPR system and shut it down, preventing bacteria from launching an immune attack. It’s the first time researchers have seen pure RNA—not protein—used to block CRISPR. In the second study [Hu H *et al. Science* 2025; eads4639], the team discovered that a special enzyme in bacteria, called DRT9, makes long strands of DNA from viral RNA. These DNA strands act like molecular weapons, helping the bacteria disrupt the virus. But viruses can also mutate to escape this defense, showing the ongoing evolutionary arms race between microbes and their viral enemies.

**Figure:**
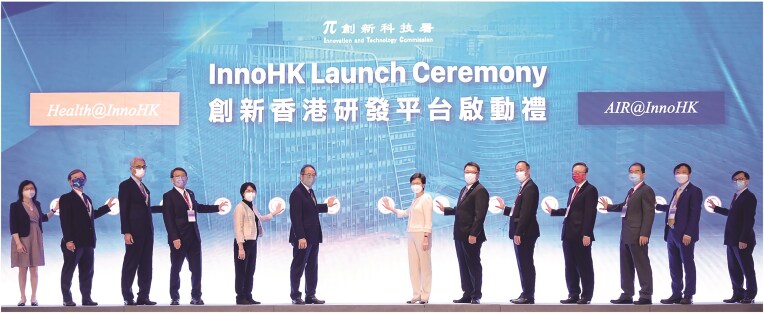
A group of university presidents and scientists launched the InnoHK initiative in Hong Kong in 2020 (Source: Hong Kong Polytechnic University).

SUSTech isn’t the only emerging scientific power in the GBA. By the end of 2024, Guangdong Province has hosted five of the ten Sino-foreign joint-venture universities on the Chinese mainland such as the Chinese University of Hong Kong, Shenzhen (CUHK–Shenzhen). New research universities such as Great Bay University (GBU) in Dongguan are mushrooming too.

Hong Kong wouldn’t lag. The government of the Special Administrative Region launched the InnoHK initiative in 2020, focusing on health science, AI and robotics technologies and promoting partnerships between leading global universities—including Oxford, Imperial College London, and the Karolinska Institute—as well as local institutions. As of mid-2025, over 30 research centers have been launched under this scheme, with a combined investment exceeding HK$10 billion. Notably, the Institute for Chinese Medicine Innovation and the Institute of Microelectronics and Semiconductor Systems will be established with direct government support, focusing on national priorities such as aging, drug development and advanced manufacturing.

‘No doubt these new universities and institutions are bringing new momentums for scientific progresses in the GBA,’ says Yanbo Wang, an associate professor of innovation policy at the University of Hong Kong (HKU).

## HUGE FUNDING SPREE

According to Wang, a solid financial foundation has been key to the GBA’s scientific ascent. In the late 2000s, the Shenzhen government made a bold move by establishing SUSTech, pledging generous research funding and salaries on par with those offered in the United States and Europe. That commitment was backed by Shenzhen’s substantial R&D investment, which reached 223.66 billion yuan (US$31.18 billion) in 2023—amounting to 6.46% of the city’s GDP.

Wei Lu, a chemistry professor and the first faculty member in his discipline at SUSTech, recalled that in the university’s early days, young principal investigators (PIs) threw themselves into research, motivated by competitive pay, ample funding and academic independence. ‘We didn’t have much equipment back then,’ he said, ‘so we borrowed time on instruments from nearby labs, such as those at Tsinghua Shenzhen Graduate School.’

**Figure:**
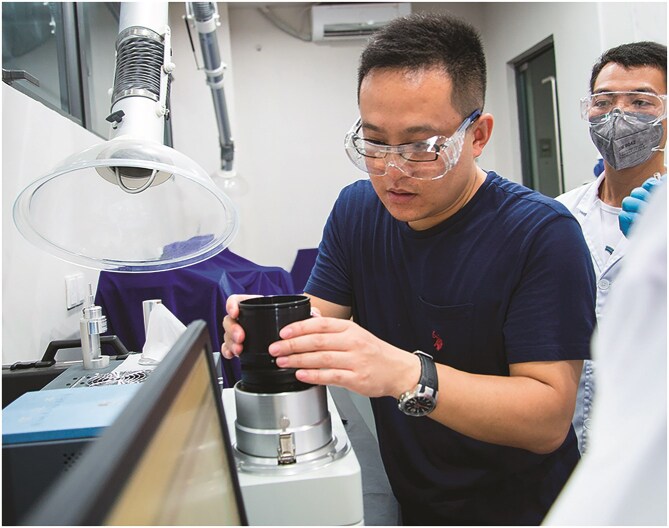
Wei Lu, a chemistry professor at SUSTech, guided his students in his lab. Lu is the one of the earliest faculty members of the young universities (Source: Wei Lu).

The university even hired an administrative staff for each PI, most returning from overseas, to deal with administration and logistics issues the PIs were unfamiliar with, so that they could concentrate on their research.

Efforts to develop major research facilities—an area where the GBA has long trailed Beijing, Shanghai, and even cities such as Lanzhou and Hefei—are now accelerating. In Shenzhen’s Guangming Science City, nine large-scale scientific installations are under construction, targeting cutting-edge fields including synthetic biology, brain simulation, materials genomics and precision medical imaging.

Wang noted that the most effective strategy adopted by GBA universities has been their ability to attract internationally renowned scientists. ‘They can quickly energize an emerging research area while mentoring and inspiring young researchers,’ he said.

In the past 5 years, a group of prestigious scientists relocated to the GBA, such as Yitang Zhang, a famous mathematician from the University of California, Santa Barbara; Nieng Yan, a structural biologist from Princeton University; Efim Zelmanov, a mathematician from the University of California, San Diego; Qiang Xu, a material scientist and chief scientist from the Japanese National Institute of Advanced Industrial Science and Technology (AIST); and Charles M. Lieber, a world renowned nano-scientist from Harvard.

SUSTech isn’t the only primary beneficiary. Shenzhen University, the newly launched GBU, and the Shenzhen Medical Academy of Research and Translation (SMART) have all attracted a group of internationally recognized science leaders.

SMART is among a new wave of non-university institutions in the GBA that are increasingly driving both scientific discovery and translational breakthroughs. A standout example is the Shenzhen Bay Laboratory (SZBL), founded in 2019 and designated as the nucleus of a future national laboratory for the life sciences. With its focus on synthetic biology, molecular design, and advanced imaging, SZBL has drawn leading scientists from around the world and aspires to become China’s counterpart to institutions such as the Broad Institute in Boston or the European Molecular Biology Laboratory headquartered in Heidelberg, Germany.

Established in 2023 with structural biologist Nieng Yan as its founding director, SMART is designed to serve as a ‘fast lane’ for medical discovery. Unlike traditional universities, it recruits global talent, operates under flexible funding mechanisms, and targets high-impact, cross-disciplinary challenges in translational medicine and cell therapy. Together, these non-university research platforms—complemented by the mega-facilities under construction in Guangming Science City—signal a profound shift in China’s innovation paradigm: from campus-centered research toward mission-driven, independently operated national research infrastructures.

**Figure:**
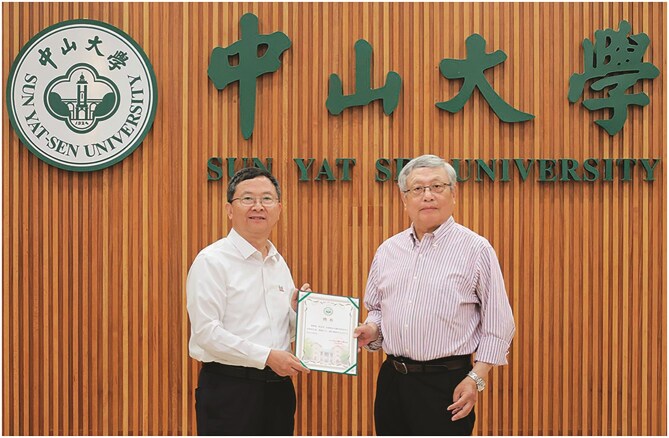
Yitang Zhang (right), a famous mathematician, received the appointment letter from SYSU president Song Gao to work at the SYSU Hong Kong Institute for Advanced Study (Source: SYSU).

## FLEXIBLE RESEARCH ENVIRONMENT

While sufficient funding level in the GBA has ensured that its research institutions attract talent, it is the flexible research environment, partly thanks to the close-to-exemplary role of Hong Kong and Macao, that helps these talents remain productive and contribute to high-quality research.

‘Unlike in Beijing and Shanghai, research institutions are very young and young scientists here are not required to follow many norms or work under leading scientists’ umbrella,’ says Gu.

That flexibility, however, depends heavily on support from universities in Hong Kong and Macao. According to Wang, institutions in the two special administrative regions can allocate postgraduate enrollment quotas under their own names, in exchange for financial collaboration with partner institutions in neighboring Guangdong Province. This arrangement enables emerging universities such as SUSTech, as well as established ones like Shenzhen University that traditionally lacked doctoral programs authorized by China’s Ministry of Education, to maintain a stable and high-caliber pool of laboratory researchers.

The resources in Hong Kong and Macau are not limited to doctoral students. In major cities of the GBA, particularly Shenzhen and Hong Kong, small-scale academic meetings and collaborations between individual labs occur almost daily. The frequent exchange sparked novel research ideas and more pragmatic evaluation, says Ye Qi, a chair professor of climate policy and dean of the society hub at Hong Kong University of Science and Technology Guangzhou (HKUST Guangzhou).

The collaboration isn’t limited to academic exchanges. The Lok Ma Chau Loop—part of the Northern Metropolis initiative—is a landmark Hong Kong–Shenzhen collaboration aimed at transforming the border area into an international innovation and technology hub.

This 87-hectare site, also known as the Hong Kong–Shenzhen Innovation and Technology Park (HSITP), is designed to integrate seamlessly with Shenzhen’s Futian Science and Technology Zone. By mid-2025, foundational infrastructure for the HSITP had been largely completed, and tenancy agreements were signed with institutions including the Hong Kong Science and Technology Parks Corporation, major Shenzhen research centers and AI-focused startups. The zone will host joint research institutes, translational labs and accelerator programs.

**Figure:**
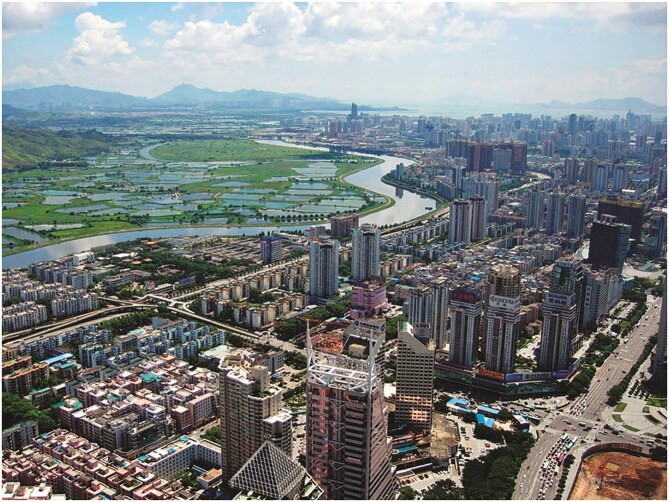
Lok Ma Chau Loop area viewed from Shenzhen (Source: SSDPenguin/Wikipedia, CC BY 3.0).

## CULTURE MATTERS

Besides decent funding and a flexible research environment, the pragmatic and collaborative culture in the Pearl River area also contributes to GBA’s rise. Guojun Shi, a senior research fellow at the Third Affiliated Hospital of SYSU in Guangzhou, notes that although his funding and research environment may not be sufficient to tackle mega science issues, his studies are fruitful in addressing clinical questions promptly.

‘People here are very pragmatic, often don’t follow those trendy hot topics but try to tap practical questions,’ says Shi, who came to GBA after having postdoc research at several leading universities in the United States.

According to Shi, scientists in Guangdong tend to communicate openly, and once they identify a shared research interest, collaboration often follows quickly. Concerns about intellectual property are minimal, he noted, as ‘people rarely steal each other’s ideas.’

‘The collaboration here is also very practical,’ Shi adds. ‘Before we start a project, everyone is upfront about the terms. No one holds back to save face, and nobody ends up shortchanged. It makes working together easy and relaxed,’ says Shi.

Both Shi and Gu now devote considerable energy to exploring the mechanism of traditional Chinese medicine (TCM) from the perspective of modern genetic sciences. Their studies are not part of any major projects to ‘modernize’ TCM, but are purely for their own interest. ‘Cantonese are enthusiastic in TCM and there should be something interesting to tap,’ says Shi.

Gu, on the other hand, links his mitochondria studies with basic TCM concepts. In a groundbreaking review article, Gu and colleagues proposed that the ancient Chinese medicine concept of ‘Yang Qi’ (阳气)—the vital life energy—shares striking biological parallels with mitochondria [Luo J *et al. Phenomics* 2022; **2**: 336–48]. The team draws detailed comparisons between the two, showing how both are essential for maintaining body temperature, energy levels, immune defense and healthy aging. The paper suggests that ‘Yang Qi’ may, in fact, reflect underlying mitochondrial health and function. The authors argue this convergence opens exciting new doors for bridging Eastern and Western medical traditions, especially in understanding complex chronic diseases like cancer, fatigue syndromes, neurodegeneration and metabolic disorders.

## SUSTAINABLE FUTURE

While the GBA has made remarkable progress, questions have been raised about its long-term sustainability. ‘Some people wonder whether the rapid rise of scientific research in the Greater Bay Area is simply the result of deep pockets—that it can lure big names and boost output quickly,’ says Wang of HKU. ‘The truth is, funding matters, but those big names have already fostered the growth of brilliant young scientists.’

A group of young leading scientists has emerged in GBA institutions. For example, over the past 5 years, 16 scientists at SUSTech have been awarded the prestigious NSFC (National Natural Science Foundation of China) Distinguished Young Scholar Grant, surpassing the number at many traditional leading research universities.

Another concern is about the insufficient synergies in funding and research management between the Chinese mainland, Hong Kong and Macao. Although Hong Kong’s Research Grants Council and the NSFC’s joint programs fund cross-border projects, most of the high-impact, visible achievements are not a result of cross-border joint funding.

However, Qi of HKUST Guangzhou does not think that insufficient joint funding is a big problem. Hong Kong and the Chinese mainland have different environments and management systems. Forcing individuals to work together under one grant often requires overcoming many procedural obstacles. ‘The key is to communicate frequently instead of always doing research of the same project together.’

Wang echoes this sentiment with his perspective: ‘Given the increasing political tension between China and the Western world, the less integration between Hong Kong and Macao and their mainland GBA partners are a good thing.’ He further explained that many world’s renowned scientists can visit and work in Hong Kong without worrying about political pressure too much. ‘Hong Kong and Macao can play as a bridge between mainland universities in the GBA and the Western world.’

Hepeng Jia is a professor of science communication at Soochow University and a freelance science writer for NSR

